# Complete Response With Cetuximab-Based Treatment of Metastatic Colorectal Cancers: Two Case Reports and Literature Review

**DOI:** 10.3389/fonc.2022.798515

**Published:** 2022-02-16

**Authors:** Li Wei, Zexiao Lin, Sidong Xie, Danyun Ruan, Wen Jiang, Yueli Cui, Sisi Liu, Tiantian Wang, Zhanhong Chen, Qu Lin

**Affiliations:** ^1^ Department of Oncology, The Third Affiliated Hospital of Sun Yat-sen University, Guangzhou, China; ^2^ Department of Radiology, The Third Affiliated Hospital of Sun Yat-sen University, Guangzhou, China; ^3^ Department of Research and Development, Nanjing Geneseeq Technology Inc., Nanjing, China

**Keywords:** cetuximab, colorectal cancer, liver metastasis, targeted therapy, complete response

## Abstract

Metastases typically develop before diagnosis and during the treatment of colorectal cancers, while patients with metastatic colorectal cancers (mCRCs) currently have a poor prognosis. In terms of surgical approaches, adjuvant therapies, and targeted therapies, the treatment of mCRCs has had numerous recent advances. As a targeted agent widely used in mCRCs, cetuximab-based treatment is still under dispute due to its side effects and unstable effect. We present two mCRC cases treated with cetuximab-based therapy, of which two patients achieved complete response and without recurrence for over 22 and 84 months, respectively. To better understand the drug usage, we also reviewed the recent achievements and usage precautions of cetuximab in mCRCs. Present and many previous observations support that cetuximab might be a referred drug in the first-line chemotherapy of mCRCs with wild-type *RAS* and *BRAF* and proficient mismatch repair.

## Background

Colorectal cancer (CRC) is the development of cancer from the colon or rectum, which has been the third leading cause of cancer-related death in both genders worldwide in 2020 (1). Over 20% of CRC patients have developed metastatic disease at the time of diagnosis, while the liver is the most common site of distant metastases (2). Currently, liver metastasis has become the leading cause of the death of CRC patients, whose 5-year overall survival rate was only ~5% (3). Surgery with adjuvant chemotherapy is the preferred approach for the treatment of CRCs, while targeted therapy is applied when necessary (4).

As epidermal growth factor receptor gene (*EGFR*) was typically overexpressed in CRCs, anti-EGFR agents, such as cetuximab and panitumumab, were commonly used as adjuvants to CRC chemotherapies (5, 6). Nevertheless, the effect of cetuximab in the first-line chemotherapy of CRCs is still under dispute, even though for patients with wild-type (WT) *RAS* and *BRAF* (7–10). We described here two cases of which patients with multiple liver metastatic sigmoid colon cancer and with liver metastasis after the comprehensive treatment of rectal cancer benefitted from cetuximab massively. For better understanding the advantages and limitations of cetuximab-based treatment, related literatures were reviewed in this report as well.

## Case Presentation

### Case 1

**Figure 1:**
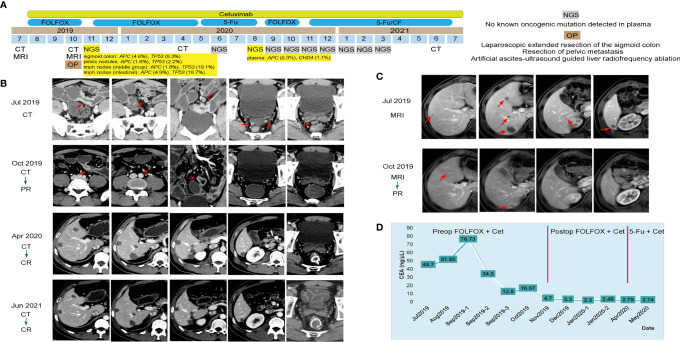
Tumor progression of the patient in case 1. **(A)** The timeline of diagnosis and treatment. **(B)** Tumors shrank and disappeared during the course of treatment by computed tomography scans of the patient’s abdomen and pelvis; tumors are indicated by red arrows. **(C)** Tumors shrank and disappeared during the course of treatment by magnetic resonance imaging of the patient’s abdomen; tumors are indicated by red arrows. **(D)** Line chart showing the changes in the levels of carcinoembryonic antigen during the course of treatment.

A 43-year-old man came to our hospital and complained of abdominal pain and constipate lasting for a week in July 2019. Blood test showed a high level of serum carcinoembryonic antigen (CEA) at 45.70 ng/ml and a normal carbohydrate antigen 19-9 (CA19-9) level at 36.73 U/ml. Colonoscopy result indicated a swelling lesion at approximately 55 cm from the anus. Pathogenic biopsy of the lesion suggested that it was a moderately differentiated adenocarcinoma. Computed tomography (CT) and magnetic resonance imaging (MRI) of the abdomen indicated sigmoid colon cancer, which involved the entire intestinal wall and metastasized in the liver and multiple lymph nodes (**Figures 1B, C**). Besides, targeted next-generation sequencing (NGS) of 425 cancer-related genes with the circulating tumor DNA (ctDNA) from plasma and tumor tissues from related lymph nodes and pelvic nodules revealed the mutation of genes *APC* c.1450G>T and *TP53* c.524G>A at a relatively high mutant allele frequency (1.6%–4.9% and 2.2%–19.1%, respectively) (**Figure 1A**). Simultaneously, the tumor was proved to be WT *RAS* and *BRAF* with proficient mismatch repair (pMMR). In light of this evidence, the final diagnosis of this patient was metastatic colon cancer at a clinical staging of cT4NxM1a (WT for *RAS* and *BRAF*).

The Eastern Cancer Cooperative Group (ECOG) performance status score of this patient was 1, which indicated the feasibility of chemotherapy. According to the National Comprehensive Cancer Network (NCCN) guidelines, the patient was initially treated with folinic acid, 5-fluorouracil and oxaliplatin (FOLFOX) plus cetuximab (six cycles) as the neoadjuvant chemotherapy. Three months later, CT and MRI results indicated the tumor and liver metastases shrank significantly in size, and his serum CEA level decreased to 16.97 ng/ml (**Figures 1B**–**D**). Therefore, comprehensive operation was implemented in October 2019, including the laparoscopic-extended resection of the sigmoid colon, the resection of pelvic metastasis, and the artificial ascites ultrasound-guided liver radiofrequency ablation. The postoperative pathological stage of the swelling lesion from sigmoid colon was diagnosed as ypT3N2bM1a. The patient subsequently underwent FOLFOX plus cetuximab (six cycles) and 5-fluorouracil (5Fu) plus cetuximab (seven cycles) as the postoperative chemotherapy consecutively. His serum CEA level dropped to a normal range (<5 ng/ml) from December 2019 (**Figure 1D**). As oncogenic mutations of *APC* c.1450G>T and *CHD4* c.91C>T were observed from the patient’s plasma by a follow-up NGS test in August 2020, the chemotherapy regimen was changed back to FOLFOX plus cetuximab (4 cycles), and the targeted NGS tests were implemented monthly for the following 7 months **(** **Figure 1A**). Consequently, no known other oncogenic mutations were detected in the following tests, thus 5Fu/CF (5-fluorouracil and folinic acid) plus cetuximab treatment was started since November 2020 (**Figure 1A**). With continuous 5Fu/CF plus cetuximab treatment (8 cycles in total), his disease maintained complete response (CR), as proved by CT scans in June 2021, and cetuximab monotherapy was started since July 2021 (**Figures 1A, B**).

### Case 2

**Figure 2:**
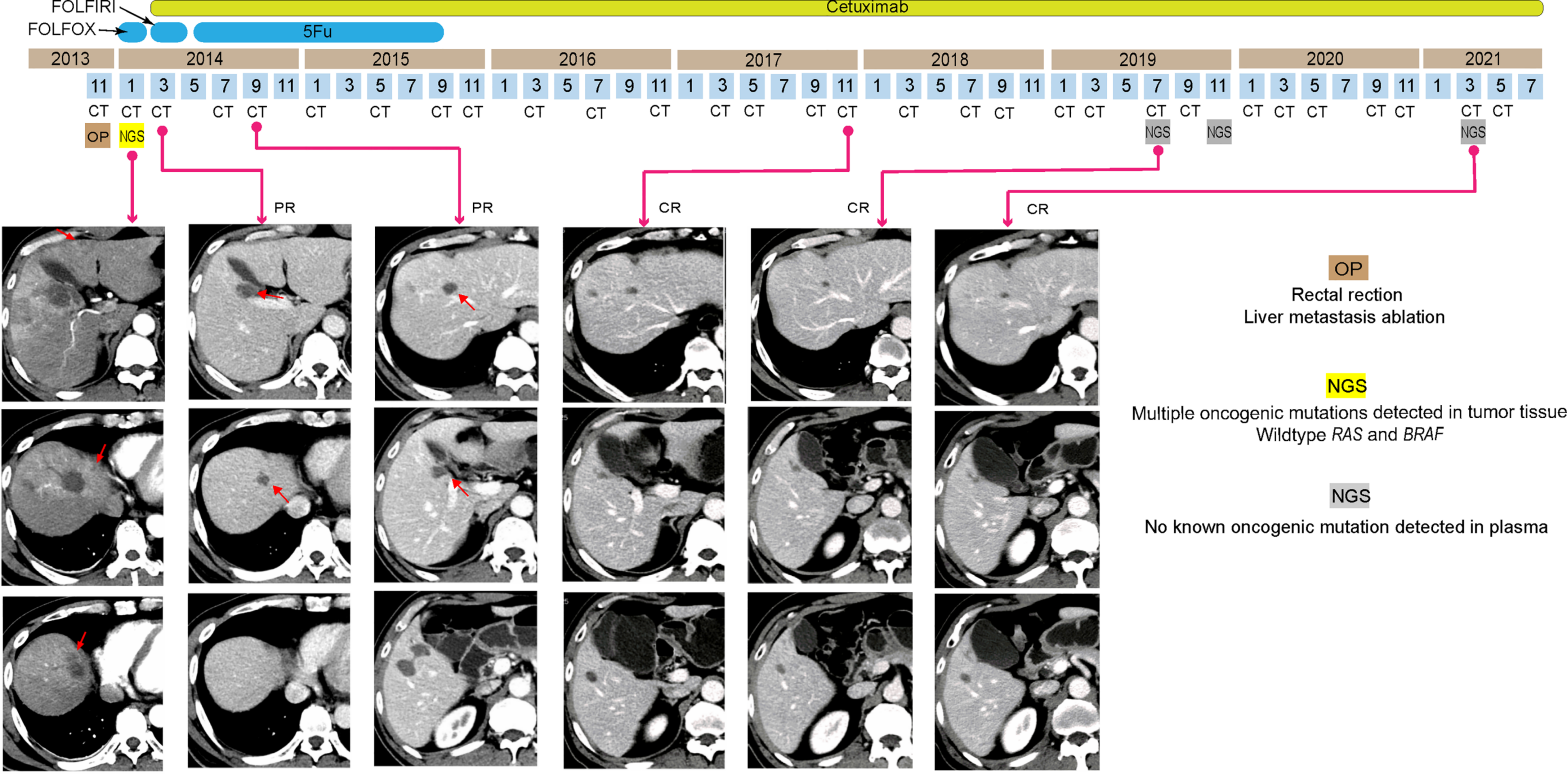
Tumor progression of the patient in case 2. Computed tomography images indicated the shrinkage and disappearance of the hepatic nodules (indicated by red arrows). PR, partial respond; CR, complete respond.

A 56-year-old man went to a hospital due to increased bowel movement and occasional blood stools in December 2013. The initial imaging evaluation with CT indicated rectal cancer with multiple liver and mesangial lymph node metastases. After radical resection in that hospital, his pathogenic biopsy revealed poorly differentiated tubular adenocarcinoma in the rectum (cT3N1M1). NGS-based genetic test of 425 cancer-related genes revealed multiple oncogenic gene mutation at high frequencies in the patient’s rectal tumor tissues (**Table 1**) including *APC* c.3929dupA and *TP53* c.572_574delCTC. Meanwhile, the tumor was proved to be *RAS* and *BRAF* WT and pMMR *via* NGS. Subsequently, the patient received 2 cycles of FOLFOX chemotherapy and radiofrequency ablation twice of liver metastases in 3 months (**Figure 2**). Afterward, he went to our hospital for further examination and recommendations in February 2014. The patient’s serum CEA, CA19-9, and cancer antigen 125 (CA125) levels were all normal, but CT images showed multiple nodules in his liver S4 and S4/8, some of which were located around the original ablation site (**Figure 2**). Thus, his final diagnosis was recurrence of rectal cancer and multiple liver metastases after the comprehensive treatments (WT for *RAS* and *BRAF*).

**Table 1:** Genetic alternations in the rectal tumor tissues of the patient in case 2.

Genes	Alternations	Coding change	Mutant allele frequency
*APC*	p.I1211Dfs*4	c.3929dupA	32.7%
*AXIN2*	p.C9Pfs*9	c.25_29delTGCCT	16.8%
*DICER1*	p.R137I	c.410G>T	21.4%
*FBXW7*	p.S582L	c.1745C>T	22.1%
*FBXW7*	p.E100*	c.298G>T	17.2%
*KMT2B*	p.R1477Q	c.4430G>A	31.2%
*LRP1B*	p.F2957L	c.8871C>A	22.4%
*TP53*	p.P191del	c.572_574delCTC	29.3%

As the patient’s ECOG status was 1, he underwent 12 cycles of folinic acid, 5-fluorouracil, and irinotecan (FOLFIRI) plus cetuximab as the second-line chemotherapy. Only mild rash was observed due to the side effect of cetuximab. CT images showed his liver metastases completely disappeared after only 2 months (**Figure 2**). Following 5-fluorouracil (5-Fu) plus cetuximab (12 cycles) and continuous cetuximab monotherapy, no oncogenic mutation was detected in the ctDNA of the patient’s plasma by NGS analysis, and no tumor was revealed by CT images from November 2017 to March 2021 (**Figure 2**). Hence, his disease had been CR for over 84 months.

## Discussion

Based on the NCCN guidelines, both FOLFOX and FORFIRI are recommended regimens in the first-line chemotherapy of CRCs, while targeted agents, such as bevacizumab and cetuximab, are allowed as additive to these regimens. In the present two cases, cetuximab was used as adjuncts in the neoadjuvant chemotherapy (FOLFOX), first-line chemotherapies (FOLFOX and 5Fu), and second-line chemotherapies (FORFIRI and 5Fu/CF), and a monotherapy drug. During the cetuximab treatment, both primary cancer and liver metastases responded remarkably. Moreover, under continuous cetuximab therapy, metastatic rectal cancer kept CR for an extremely long time (over 7 years) in case 2. Due to the concerns of recurrence and little economic burden, both patients preferred to be treated with cetuximab for the rest of their life.

Cetuximab performed effectively in previous metastatic CRC cases as well, including liver, lung, bone, urinary system, and various lymph node metastases (**Table 2**). Aside from the current chemotherapy regimens, cetuximab could also play critical roles in the regimens of FOLFOXIRI (folinic acid, 5-fluorouracil, oxaliplatin, and irinotecan), ZOL (zoledronic acid), and irinotecan (**Table 2**). In addition, bevacizumab, vascular endothelial growth factor targeted agent in the treatment of CRCs, was proved to be less effective than cetuximab by statistic studies, especially when the tumor developed in the left-sided colon (20–22). However, panitumumab, another recommended anti-EGFR agent for CRCs, could be an alternative to cetuximab, as they effect equivalently (23). In view of the above evidence, cetuximab might be a preferred drug when treating metastatic CRCs.

**Table 2:** Metastatic colorectal cancer cases benefitted from cetuximab-based treatment.

Reference	Age (y), gender	Distant metastatic site	Cet-based treatment	Response	FUS (m)
Qiu et al. (11)	51, M	Liver	Neo: FOLFOXIRI + cet; 1st: FOLFOX + cet	PR	7
Li et al. (12)	52, M	Liver and lung	3rd: cet + fruquintinib	SD	8
Tokumaru et al. (13)	58, M	Bone	3rd: ZOL + cet	PR	3
Cohen et al. (14)	20, M	Liver	3rd: irinotecan + cet	CR	131
Lu et al. (15)	90, F	Bladder, kidney, and ureter	FOLFIRI + cet without surgery	SD	20
Van et al. (16)	43, F	Liver and lung	1st: FOLFIRI + cet	CR	33
Schoellhammer et al. (17)	68, F	Liver	Neo: FOLFOX + cet	PR	30
Chang and Huang (18)	64, M	Liver, lung, and bone	3rd: FOLFIRI + cet	SD	7
Murono et al. (19)	63, M	Lung and kidney	4th: cet monotherapy	CR	12
Current case 1	43, M	Liver	Neo and 1st: FOLFOX + cet	CR	22
Current case 2	56, M	Liver	2nd: FOLFIRI + cet	CR	86

M, male; F, female; Neo, neoadjuvant chemotherapy; 1st, 2nd, 3rd, and 4th, first-, second-, third-, and fourth-line adjuvant chemotherapy, respectively; Cet, cetuximab; FOLFOXIRI, folinic acid, 5-fluorouracil, oxaliplatin and irinotecan; ZOL, zoledronic acid; PR, partial respond; CR, complete respond; SD, stable disease; FUS, follow-up survival.

Despite these achievements, cetuximab along with other anti-EGFR drugs could also bring severe side effects in the CRC treatments, sometimes even life threatening (23). Rash is the most common side effect of cetuximab due to its skin toxicity, which has been observed in over 60% of the related cases (24). Moreover, some rare side effects have also been reported occasionally, such as consciousness lost, interstitial pneumonitis, and subcutaneous abscess (25–27). In our cases, both patients were well tolerated to cetuximab, while only mild rash was observed in case 2.

In the CRC first-line chemotherapy, anti-EGFR monoclonal antibodies have been clinically and statistically confirmed less effective when treating patients with *RAS* and *BRAF* mutations (7, 8, 28, 29). However, quite a few studies supported that cetuximab might be effective in *KRAS* G13D mutation (accounts for approximate 16% of all *KRAS* mutations) (30, 31). In terms of the microsatellite instability, a recent study implied that cetuximab could even promote disease progression in stage III colon cancer patients with deficient mismatch repairing (5). Thus, the detection of tumor-related genes is necessary prior to the usage of cetuximab, especially for those in mitogen-activated protein kinase (MAPK) signal pathways. Patients in the presented cases were all confirmed WT *RAS* and *BRAF*, and pMMR by NGS analysis of both tumors and plasma ctDNA before treatments.

As previous clinical trials generated dramatically contrast outcomes, the effect of cetuximab in the first-line chemotherapy of CRCs is still under controversy. On the one hand, comparative analysis of FOLFIRI with or without cetuximab of mCRC-treatment suggested that cetuximab could reduce the risk of disease progression (32). Moreover, significant improvement was observed in the overall response rates of mCRC treated with FOLFOX plus cetuximab than FOLFOX alone (28). On the other hand, both in North America and Europe, statistical studies of patients with resected stage III colon cancer indicated that there was no improvement of disease-free survival when adding cetuximab to the regimen FOLFOX (9, 33). Besides, addition of cetuximab to chemotherapy and surgery for operable colorectal liver metastases could even result in shorter progression-free survival (10). Therefore, more clinical cases and trials should be conducted for further validations of cetuximab-related treatment in CRCs, whereas our two cases obviously stand at its positive side.

## Conclusion

In this report, we described two metastatic CRC cases that benefitted from cetuximab and reviewed its recent success and usage precautions. Data from the present and previous cases indicate both primary and metastatic tumors respond quickly to this anti-EGFR agent, however, only for those with WT *RAS* and *BRAF* and pMMR. By-effects after the treatment of cetuximab are varied largely from individuals, which implies the development of predictive biomarkers associated with the sensitivity of cetuximab and other anti-EGFR drugs.

## Data Availability Statement

The original contributions presented in the study are included in the article/supplementary material. Further inquiries can be directed to the corresponding authors.

## Ethics Statement

The studies involving human participants were reviewed and approved by Ethical Committee of The Third Affiliated Hospital of Sun Yat-sen University. The patients/participants provided their written informed consent to participate in this study.

## Author Contributions

LW, ZL, and SX contributed equally to this report. All authors prepared the manuscript. TW, ZC, and QL designed the clinical treatment for the patients. LW, ZL, and DR were in charge of diagnosis. LW and ZL were in charge of patient care and performed all surgical procedures. All authors contributed to the article and approved the submitted version.

## Funding

This study was supported by the Natural Science Foundation of Guangdong Province (project number: 2018A0303130282).

## Conflict of Interest

WJ, YC, and SL are employees of Nanjing Geneseeq Technology Inc., China.

The remaining authors declare that the research was conducted in the absence of any commercial or financial relationships that could be construed as a potential conflict of interest.

The reviewers MQ and ZL declared a shared parent affiliation with several of the authors LW, ZL, SX, DR, TW, ZC, and QL, to the handling editor at the time of review.

## Publisher’s Note

All claims expressed in this article are solely those of the authors and do not necessarily represent those of their affiliated organizations, or those of the publisher, the editors and the reviewers. Any product that may be evaluated in this article, or claim that may be made by its manufacturer, is not guaranteed or endorsed by the publisher.
